# Residue Depletion of Florfenicol and Florfenicol Amine in Broiler Chicken Claws and a Comparison of Their Concentrations in Edible Tissues Using LC–MS/MS

**DOI:** 10.3390/molecules23092211

**Published:** 2018-08-31

**Authors:** Ekaterina Pokrant, Ricardo Riquelme, Aldo Maddaleno, Betty San Martín, Javiera Cornejo

**Affiliations:** 1 Department of Preventive Medicine, Faculty of Veterinary and Animal Sciences, University of Chile, Av. Santa Rosa, 11735 La Pintana, Santiago, Chile; katiavalerievna@ug.uchile.cl (E.P.); lia@veterinaria.uchile.cl (R.R.); 2 Laboratory of Veterinary Pharmacology, Faculty of Veterinary and Animal Sciences, University of Chile, Av. Santa Rosa, 11735 La Pintana, Santiago, Chile; amaddaleno@veterinaria.uchile.cl (A.M.); bsmartin@uchile.cl (B.S.M.)

**Keywords:** florfenicol, florfenicol amine, antimicrobial residues, muscle, liver, chicken claws, LC–MS/MS

## Abstract

Antimicrobial residues might persist in products and by-products destined for human or animal consumption. Studies exploring the depletion behavior of florfenicol residues in broiler chicken claws are scarce, even though claws can enter the food chain directly or indirectly. Hence, this study intended to assess the concentrations of florfenicol (FF) and florfenicol amine (FFA)—its active metabolite—in chicken claws from birds that were treated with a therapeutic dose of florfenicol. Furthermore, concentrations of these analytes in this matrix were compared with their concentrations in edible tissues at each sampling point. A group of 70 broiler chickens were raised under controlled conditions and used to assess residue depletion. Sampling points were on days 5, 10, 20, 25, 30, 35, and 40 after ceasing treatment, thus extending beyond the withdrawal period established for muscle tissue (30 days). Analytes were extracted using HPLC-grade water and acetone, and dichloromethane was used for the clean-up stage. Liquid chromatography coupled to mass spectroscopy detection (LC–MS/MS) was used to detect and quantify the analytes. The analytical methodology developed in this study was validated in-house and based on the recommendations described in the Commission Decision 2002/657/EC from the European Union. Analyte concentrations were calculated by linear regression analysis of calibration curves that were fortified using an internal standard of chloramphenicol-d_5_ (CAF-d_5_). The depletion time of FF and FFA was set at 74 days in claws, based on a 95% confidence level and using the limit of detection (LOD) as the cut-off point. Our findings show that FF and FFA can be found in chicken claws at higher concentrations than in muscle and liver samples at each sampling point.

## 1. Introduction

One of the distinguishing features from the poultry industry in recent decades has certainly been the increasing production volumes to keep pace with the demands from a growing population. Furthermore, chicken meat has become a source of animal protein that is readily available and economically affordable, unlike products sourced from other animal species. According to the Food and Agriculture Organization (FAO) [1], chicken meat accounted for 36.5% of all meat produced worldwide (321.3 million metric tons), which is equivalent to 117.2 million metric tons of chicken meat, globally. Similarly, the United States Department of Agriculture (USDA) documented that, in 2017, chicken meat production increased consistently up to 18,696 metric tons in the US [2].

Poultry by-products can be regarded either as raw material or as products with a greater added value [3]. Among them, the most relevant are meals, such as those made from bones, feathers, or blood. These by-products are used for different purposes, such as feeding other animal species, or even as farming fertilizers [4]. Chicken claws are also poultry by-products, and they have increased in production strongly over recent years. In fact, the United States exported 2.72 million metric tons of chicken claws only in the year 2013 [5]. Chile reported an exportation volume of 14,990 tons of chicken claws on 2014 [6]. The destination for these products was mainly the Asian market, especially China.

In poultry farming, antimicrobials are used for the treatment of bacterial diseases, thus leading to the publication of several studies about the presence of antimicrobial residues in animal by-products. For example, reports from various authors [7,8,9], showed that residues of sulfachloropyridazine, fluoroquinolones, oxytetracycline, florfenicol, and tylosine are transferred and deposited in chicken feathers. In these works, researchers found that drug concentrations persisted in feathers for longer periods than in common edible tissues, even after meeting the withdrawal periods that are established for edible tissues when using commercial drug formulations.

Meanwhile, studies examining bioaccumulation of veterinary drugs in chicken claws are scarce. One of them [10] explored the behavior of oxytetracycline (OTC) and its active metabolite (4-epi-oxytetracycline) in broiler chicken claws treated with a therapeutic dose of 10% OTC. By day 3 after ceasing treatment, they quantified concentrations of 2997 µg·kg^−1^ for both OTC and 4-epi-OTC. These researchers were also able to detect these analytes even at day 19, thereby calculating a withdrawal period of 50 days, for a 95% confidence level.

Considering that this food product—which includes all foot structures below the spur—has become a highly profitable export by-product for the broiler chicken industry, the presence of antimicrobial residues in them must be observed [11]. Chicken claws can be destined either for direct consumption or for rendering [12], as they can be prepared as a main course meal, a snack, or even used to extract jelly from them [13,14]. It is, therefore, highly relevant to study the behavior of antimicrobials commonly used in poultry farming, in regards to their transference profiles to these parts of the animals too.

Florfenicol (FF) is a synthetic, broad-spectrum antimicrobial, and it is one of the most commonly used drugs in poultry farming operations, as it was specifically designed for veterinary use [15]. It is active at lower concentrations than thiamphenicol—from which it is derived—and chloramphenicol, both drugs that are its structural analogs [16]. Its spectrum of activity makes it effective against enteric bacteria, such as *Enterobacter cloacae*, *Shigella dysenteriae*, *Salmonella typhi*, *Klebsiella pneumoniae*, *Staphylococcus aureus*, *Pasteurella multocida*, *Proteus vulgaris*, and *Escherichia coli* [17]. Florfenicol is metabolized to florfenicol amine (FFA), florfenicol oxamic acid, and florfenicol alcohol. Though the rate among them varies between animal species, florfenicol amine is the major metabolite in most farm animals that are destined for human consumption, which is why it is assigned the role of a marker residue of florfenicol [18].

The broad spectrum of activity of florfenicol, in addition to its therapeutic effectiveness and low risk for toxicity, makes it increasingly more important within the context of farm animals destined for human consumption [15,19,20].

The European Union established maximum residue limits (MRLs) for the sum of FF and FFA residues at concentrations of 100 μg·kg^−1^ in muscle, 200 μg·kg^−1^ in the skin and fat tissues, 750 μg·kg^−1^ in the kidney, and 2500 μg·kg^−1^ in the liver of poultry destined for human consumption [21].

Meanwhile, the Codex Alimentarius, which lays down international regulations for foodstuffs and determines maximum residue limits for veterinary drugs, has not set limits for FF and FFA in any matrix [22]. However, determining such MRLs in several farm animal species is a necessity for many member countries of the Food and Agriculture Organization, according to the global survey that informed the Codex Committee on Residues of Veterinary Drugs in Foods [23].

Some studies developed methodologies for the detection of phenicols in edible tissues of chicken. One of these methods [24] uses LC–MS/MS for the routine analysis of FF residues in this matrix and could also be applied to the analysis of liver samples. The same group of researchers adapted and revalidated a simple, short, and rapid confirmatory method for the simultaneous analysis of multi-amphenicol residues in poultry meat [25]. Bearing in mind the importance of chicken as a source of white meat globally, the development of these analytical methods is highly relevant.

However, claws are a poultry by-product; hence, they are not subject to meet an MRL, in spite of their possible role as an important vehicle that may transfer antimicrobial residues along the production line. Such an event could impact the health of consumers either via its direct consumption, or because claws are used as ingredients in diets of other farm animal species.

In light of the evidence above, this study intended to explore FF and FFA residue concentrations in broiler chicken claws, as well as determining the depletion time of these antimicrobial drugs, following the administration of a therapeutic dose of a commercial formula of 100% FF that is currently registered for veterinary use in broiler chickens. Additionally, the concentrations of these drugs were assessed in muscle and liver tissue to draw comparisons of the concentrations of these residues between these three matrices.

## 2. Results

### 2.1. In-House Validation of the Analytical Methodologies

**Figure 1:**
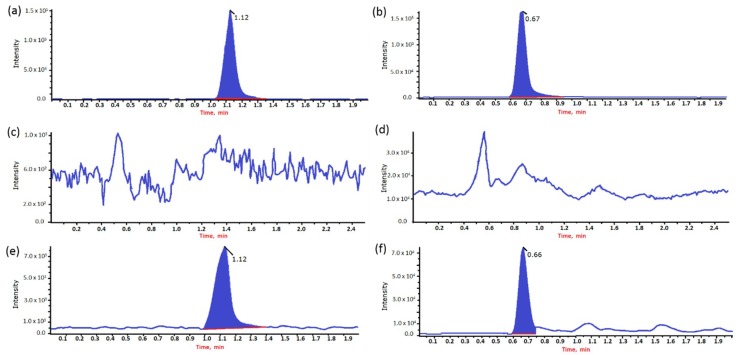
Chromatograms of florfenicol (FF) in pure standard solutions, blank samples, and samples fortified with FF and florfenicol amine (FFA) at a concentration of 50 µg·kg^−1^. (**a**) Pure standard of FF; (**b**) pure standard of FFA; (**c**) blank claw sample of FF; (**d**) blank claw sample of FFA; (**e**) chromatograms of sample fortified with FF; (**f**) chromatograms of sample fortified with FFA.

The specificity of the method was proven using LC–MS/MS analyses of blank samples of claws, muscle, and liver from broiler chickens. The retention time of analytes showed no drug interferences in blank samples (Figure 1). The limits of detection (LODs) for both analytes were set at a concentration of 50 µg·kg^−1^ for claws and 20 µg·kg^−1^ for muscle and liver. The limits of quantification (LOQs) for FF were 56.8, 25.2, and 22.1 µg·kg^−1^ for claws, muscle, and liver samples, respectively. Meanwhile, the LOQs for FFA were 53.7, 25.4, and 22.6 µg·kg^−1^ for claws, muscle, and liver samples, respectively.

Calibration curves for claws were spiked at concentrations 0, 50, 100, 200, and 400 µg·kg^−1^. Their linearity was evidenced by an average coefficient of determination (R^2^) above 0.99 for both FF and FFA. Meanwhile, the concentrations of the calibration curves for muscle and liver samples were at 0, 20, 50, 100, and 200 µg·kg^−1^. In these samples, R^2^ for FF and FFA was above 0.99 for both matrices.

The recovery of FF and FFA in claws, ranged from 88.91 to 115.83%, while, for muscle and liver samples, these ranges were 87.08 to 104.66%, and 95.35 to 112.83%, respectively.

As for the precision of the analytical method, it was expressed as the relative standard deviation (RSD) of both the repeatability and intra-laboratory reproducibility. Repeatability for FF in claw samples showed an RSD of 10.1% at the 50 µg·kg^−1^ level, and reproducibility showed an RSD of 11.1% for the same concentration. Meanwhile, repeatability for FFA showed an RSD of 19.5%, and reproducibility showed an RSD of 25.8% at the same level of fortification. In the case of muscle samples, repeatability and reproducibility RSDs were 9.0 and 23.1% for FF, and 5.9 and 16.9% for FFA, respectively. For liver samples, these RSDs showed values of 4.1 and 13.0% for FF, whereas, for FFA, the RSD values observed were 6.8 and 18.1% at a fortification concentration of 20 µg·kg^−1^.

### 2.2. Detection and Quantification of FF and FFA Concentrations in Samples of Claws, and Muscle and Liver Tissues

FF and FFA concentrations were assessed in the muscle, liver, and claws of treated birds. Residues were quantified at each sampling day using the equation of regression analysis, *y = a + bx* (where *y* is the area, *a* is the *y*-intercept, *b* is the slope, and *x* is the concentration). This regression equation was applied on calibration curves of fortified matrices, considering a determination coefficient (R^2^) ≥ 0.99. The construction of calibration curves requires using blank samples for positive and fortified controls. Thus, these samples were claws, muscle, and liver tissue that were sourced from birds in group B, and were included in every batch of samples analyzed, and at each sampling point. Table 1 lists the concentrations that were found throughout this study.

**Figure 2:**
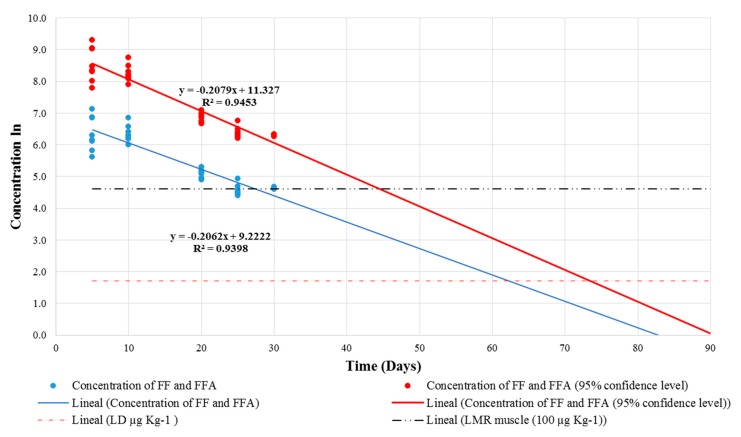
Depletion of FF and FFA residue concentrations in broiler chicken claws (95% confidence level), showing a withdrawal time of 74 days.

The withdrawal time for FF and FFA in chicken claws was calculated on the basis of the recommendations from the European Medicines Agency, which are explained in its “Guideline on Approach toward Harmonization of Withdrawal Periods”, EMA/CVMP/SWP/735325/2012 [26]. Firstly, residue concentrations were plotted using a semi-logarithmic scale of concentration against time (Figure 2). Then, concentrations were subjected to a linear regression analysis, considering a 95% confidence level. This allowed us to determine the time when residue concentrations declined below the LOD that was established for the analytical method. Hence, the withdrawal time for FF and FFA residues in chicken claws was set at 74 days.

## 3. Discussion

Before assessing residue concentrations of FF and FFA in claws, and muscle and liver samples, it was necessary to perform an in-house validation of the analytical methods to ensure their suitability for the experimental phase of this research. An internal protocol was designed for this purpose, on the basis of regulations of the European Commission [27]. The parameters that were assessed were retention time, linearity of the calibration curve, recovery, and precision. LODs and LOQs were calculated following the recommendations from the Food and Drugs Administration of the United States of America [28]. According to the results observed for the validation parameters, these methods met the acceptance criteria that were specified by the internal validation protocol. Hence, the analytical methodologies implemented in this study proved their suitability for the detection and quantification of FF and FFA analytes in claw, muscle, and liver matrices.

The sum of the concentrations of both analytes was used to calculate the withdrawal time in chicken claws, following the directive EMA/CVMP/SWP/735325/2012 [26]. The reasons for also measuring FFA concentrations were that this drug is the main metabolite of FF (i.e., it becomes a marker of the generation of metabolites in an individual) and also because it has antimicrobial activity on its own.

Others [29] found similar results for oxytetracycline. In those studies, they analyzed samples of muscle and bone tissues from broiler chickens who were treated with a therapeutic dose of a 20% oxytetracycline formulation, concluding that their bones evidenced a high bioaccumulation of oxytetracycline in them. Not long after, Cornejo et al. [10] assessed the depletion of oxytetracycline in claws from broiler chickens who received a diet containing a commercial formulation of this drug. These authors found high concentrations of oxytetracycline residues, and these drugs persisted for longer periods than the established withdrawal time for that specific formulation in muscle tissues. Such elevated concentrations could be explained by the pharmacokinetic characteristics of oxytetracycline, which is quickly and efficiently absorbed in the gastrointestinal tract, and then widely distributed to extravascular tissues. This drug penetrates the body tissues well and is also reabsorbed in the kidneys at the tubular level [15,16].

The results in this study agree with previous works and are particularly similar to those observed by Cornejo et al. [30], as the concentrations of FF and FFA persisted for a longer period of time in chicken claws than the withdrawal time currently established in muscle tissues for the commercial formulation. Furthermore, for each sampling point, residue concentrations of these analytes were detectable at greater concentrations in claws than in samples of muscle and liver tissues that were sourced from the same animals. In fact, their concentrations were greater than the MRL for muscle tissue (100 µg·kg^−1^), even when, in muscle and liver samples, these residues already declined below the LOD (20 µg·kg^−1^) previously set for the analytical method. Thus, by day 5 after ceasing treatment, an average of 651.9 µg·kg^−1^ of FF and FFA was quantified in the claw matrix. Concentrations decreased 8.4% on day 10 post-treatment. For day 30 post-treatment the concentrations quantified for FF plus FFA reached 102.4 µg kg^−1^, and were higher than on day 25 after treatment. This can be attributed to the fact that FFA concentrations in the fifth sampling point were greater than those from the fourth sampling point. These analytes bioaccumulated at high concentration levels in claws, even when FF and FFA residue concentrations in muscle and liver samples fell below the MRL. The results observed in this work showed that FF and FFA residue concentrations in claws were 10-fold greater than those in the muscle matrix, whereas concentrations in liver samples were even below the LOD established for the method. A study published by Chang et al. [15] indicates that, after a single oral dose of FF (30 mg/kg), concentrations obtained from leg muscle were significantly higher than those in breast muscle. This could be comparable with our results, since we also found higher concentrations of these analytes in claws compared with the other matrices; thus, it could be inferred that there is a greater distribution and persistence of this drug into claws. In addition, these structures have a higher concentration of lipids, which is a characteristic that also hinders the chemical extraction of residues. According to this, it was necessary to optimize the analytical methodology using a greater volume of dichloromethane to clean up claw samples, thus avoiding sample emulsion.

However, the presence of FF and FFA residues entails a risk to public health, due to the potential of contaminated products to reenter the food chain. Some of the effects of antimicrobial residues on human health include allergic reactions, carcinogenesis, and mutagenesis, and they also contribute to the development of bacterial resistance to antimicrobials. The latter concern is highly significant in light of the current efforts to promote the correct use of these drugs and thwart their overuse. Otherwise, pathogenic bacteria might be exposed to a selection pressure that could make them resistant to these products [31,32].

Consequently, it is imperative that more studies continue exploring possible reentry routes for antimicrobials into the food chain, whether it happens via this matrix or any alternative ones. Moreover, such a body of knowledge would lay the groundwork for the establishment of regulations concerning the MRLs that can be tolerated for those residues that are not already included in current directives. It would also enable the creation of a surveillance system for antimicrobial residues in broiler chicken claws, with the purpose of ensuring food safety to consumers, as well as preventing these residues from escaping into the environment.

## 4. Materials and Methods

### 4.1. Experimental Animals: Controlled and Treated Groups

The Bioethics Committee of the Faculty of Veterinary and Animal Sciences of the University of Chile granted ethical approval for the experimental design of this work (Certificate N° 23−2014). Seventy male one-day-old broiler chickens (Ross^®^ 308, Aviagen Inc., Huntsville, AL, USA) were kept in individual cages (25 ± 5 °C and 50–60% relative humidity) and provided with ad libitum access to water and non-medicated feed.

These experimental animals were raised and monitored within indoor facilities, which were provided by the Laboratory of Avian Pathology from the Faculty of Veterinary and Animal Sciences of the University of Chile.

Inspection and management protocols were designed on the basis of the Animal Protection Act N° 20,380 of the Chilean legislation [33], as well as Directive 2010/63/EU [34] on the protection of animals used for scientific purposes. The slaughter protocol was designed in conformity with the European Council Regulation (EC) No. 1099/2009 [35] on the protection of animals at the time of killing.

The size of the experimental groups for this depletion study was calculated following criteria and recommendations established by the European Medicines Agency “Guideline on Approach toward Harmonization of Withdrawal Periods”, EMA/CVMP/SWP/735325/2012 [26]. As for the treatments, group A (56 birds) was treated with 10% FF at a concentration of 30 mg·kg^−1^ of body weight. This treatment was administered orally, once a day for five consecutive days. Meanwhile, group B (14 birds) was assigned as the control group; thus, it received no treatment and it was kept under the same conditions than group A.

### 4.2. Quantification of FF and FFA in Broiler Claws

#### 4.2.1. Standard Solutions and Reagents

FF and FFA standards of 99.8% certified purity were used for the analysis and quantification of the analytes, and chloramphenicol-d_5_ (CAF-d_5_) of 97% certified purity was used for the internal standard. All standards were manufactured by Sigma Aldrich, Inc. (now Merck & Co., Darmstadt, Germany)

The spiking solutions of FF, FFA, and CAF-d_5_ were prepared in a solution of methanol/water (8/2) at a concentration of 5000 ng·mL^−1^.

Reagents such as water (LiChrosolv^®^), acetone (Emsure^®^), dichloromethane (LiChrosolv^®^), and hexane (LiChrosolv^®^), were sourced from Merck & Co., Inc., while methanol certified for use in liquid chromatography (HPLC and UHPLC) and spectrophotometry, was sourced from J.T. Baker^®^ (Avantor™ Performance Materials, Inc., Center Valley, PA, USA).

#### 4.2.2. Sample Collection and Processing

Eight birds from group A and two from group B were slaughtered at days 5, 10, 20, 25, 30, 35, and 40 after ceasing treatment. The sampling points were set at those specific dates taking into account that the withdrawal period for the chosen formulation used was 30 days.

Claws, and muscle and liver samples were collected immediately after euthanasia, and were individually stored at −20 °C in properly identified plastic bags. Claws were then washed with milli Q water (18.2 MΩ·cm at 25 °C) to ensure proper elimination of external contaminants in our samples. This procedure resembled current carcass washing practices that are routinely performed in abattoirs. Afterwards, claws were chopped with scissors and subsequently homogenized in a food processor. Meanwhile, muscle and liver samples had all their fat removed before being homogenized in the food processor. Finally, all samples were stored individually in plastic bags at −20 °C, waiting for the extraction procedure.

#### 4.2.3. Extraction Procedure

The extraction procedure is a required step to prepare samples for analysis via liquid chromatography coupled to mass spectrometry (LC–MS/MS). Our extraction methodology for FF and FFA in claw, muscle, and liver matrices was based on previous works from other authors [36,37,38], who described techniques able to detect these analytes in muscle and liver from fish, as well as in muscle, liver, and kidneys from pigs, and in muscle samples from chickens, respectively.

For the extraction of FF and FFA from chicken claws, samples of 2 ± 0.02 g were weighed in a 50-mL polypropylene tube and then spiked with 40 ng·g^−1^ of CAF-d_5_. Then, they were extracted using a solvent mixture made from 10 mL of water and 10 mL of acetone. These samples were agitated for 5 min and then centrifuged for 5 min at 4000× *g*. The supernatant was collected from the vial and subsequently transferred to another polypropylene tube. Then, 15 mL of dichloromethane was added to the supernatant before the sample was agitated again for 5 min and centrifuged for another 5 min at 4000× *g*. The upper phase of the solution was discarded.

The sample was evaporated under a mild nitrogen flux at 40–50 °C, and reconstituted later on in 700 μL of a solution of methanol/water (7/3). The reconstituted sample was mixed with 1 mL of hexane, agitated for 5 min, and centrifuged again for 5 min at 4000× *g*. Following this, 700 µL of the sample was recovered, transferred to an Eppendorf tube, and centrifuged for 10 min at 17,000× *g*. Finally, the sample was filtered through 33-mm millex filters with 0.22-µm polyvinylidene fluoride (PVDF) membranes and transferred to a glass vial.

The main difference for the extraction method of edible tissue samples was the volume of solvents required. In the case of the muscle matrix, it was 5 mL of water, and both muscle and liver samples required 5 mL of dichloromethane.

#### 4.2.4. Instrumental Analysis

An Agilent 1290 Infinity Series liquid chromatograph device coupled to a triple quadrupole API 5500 mass spectrometer (AB SCIEX, Framingham, MA, USA) was used for the instrumental analysis. Also, the Analyst^®^ version 1.6.3 and Multiquant^®^ version 3.0 software packages (AB SCIEX, Framingham, MA, USA) were used for equipment management and integration, respectively.

A Synergi^TM^ (Torrance, CA, USA) 4-μm fusion RP 80 Å 50 × 2.0 mm analytic column was used for the chromatographic separation of the analytes, using a mobile phase of two solvents. Solvent A was 0.1% acetic acid in water, and solvent B was 0.1% acetic acid in water/methanol (1/9). The gradient flow was 350 μL·min^−1^ and the gradient elution was isocratic, with 25% phase solvent A, and 75% phase solvent B. The injection volume was 2 μL, and the column oven temperature was set at 37 °C.

A multiple reaction monitoring (MRM) scan type was used for acquiring and visualizing LC–MS/MS data. The source temperature was 550 °C, and the pressure was 60 psi for the nebulizer (GS1), 80 psi for the turbo ion (GS2), 20 psi for the curtain gas, and 10 psi for the collision gas. The ionization was performed by electrospray, and the ion spray voltage was 4500 V. Table 2 lists the monitored ion masses.

### 4.3. In-House Validation of the Analytical Methods

The internal validation procedure was performed following the recommendations from the Commission Decision 2002/657/CE of the EU [27], for the parameters of retention time, linearity, recovery, and precision. The LOD was calculated as the mean of the assay results from 20 fortified control samples at 20 µg·kg^−1^, with a signal-to-noise ratio of 3:1. Meanwhile, the LOQ was set following the recommendations of the FDA VICHGL49 [39]. According to this guideline, the LOQ is calculated as the sum of the LOD plus 1.64 times the standard deviation, and was based on the results from 20 samples of each matrix fortified at the LOD.

### 4.4. Depletion Study

In order to determine the depletion time of FF and its active metabolite, FFA, in chicken claws, samples were collected at days 5, 10, 20, 25, 30, 35, and 40 after ceasing treatment. A linear regression was performed then, using the concentration results for each sampling date and following the statistical analysis recommendations from the “Guideline on Approach toward Harmonization of Withdrawal Periods”, EMA/CVMP/SWP/735325/2012 [26], published by the European Medicines Agency. This guideline describes the statistical approach that the agency recommends for establishing withdrawal periods, as well as the minimum number of sampling points and animals that are required to ensure statistical robustness. In the case of chickens, however, the required number of animals per sampling point was determined according to the guideline VICH GL48 on “Studies to Evaluate the Metabolism and Residue Kinetics of Veterinary Drugs in Food-Producing Animals: Marker Residue Depletion Studies to Establish Product Withdrawal Periods” [28]. The VICH GL48 guideline specifies that a minimum of six birds are required per sampling point for poultry depletion studies. Afterward, a depletion curve was plotted on a semi-logarithmic scale using the K. Stange equation, considering a 95% confidence level to determine withdrawal times. Bearing in mind that no MRL was set for this matrix, the LOD established for this analytical methodology was used as a cut-off point.

## 5. Conclusions

The methodologies implemented and validated in this work allowed us to detect and quantify FF and FFA residues in claw, muscle, and liver matrices. The concentrations of these residues detected in this work showed that these drugs persisted at greater concentrations in claws than in samples of muscle or liver tissues that were collected from the same individuals at each sampling point, even when these concentrations fell below the LOD (20 µg·kg^−1^).

The depletion analysis of FF and FFA described in this work shows that these drugs bioaccumulate in chicken claws, which could lead to antimicrobial residues reentering the food chain when these by-products are used in animal or human diets. Residues of FF and FFA persist in chicken claws for longer periods than in other matrices, even surpassing the average lifespan of broiler chickens (74 days).

These results show that claws from broiler chickens that are treated with this antimicrobial need to be monitored and controlled to ensure protecting the health of consumers and improving public health.

## Figures and Tables

**Table 1:** Depletion of florfenicol (FF) and florfenicol amine (FFA) in claws, muscle, and the liver of broiler chickens.

Sampling Point	Days after Ceasing Treatment	Age of Birds (Days)	Average Concentration of FF + FFA in Claws Samples (ng·g^−1^)	Average Concentration of FF + FFA in Muscle Samples (ng·g^−1^)	Average Concentration of FF + FFA in Liver Samples (ng·g^−1^)
1	5	15	651.9	68.9	<LOD
2	10	20	596.9	<LOQ	<LOD
3	20	30	161.0	<LOD	<LOD
4	25	35	97.9	-	-
5	30	40	102.4	-	-
6	35	45	<LOD	-	-
7	40	50	<LOD	-	-

FF: florfenicol; FFA: florfenicol amine; LOD: limit of detection; LOQ: limit of quantification.

**Table 2:** Monitored ion masses.

Analyte	Precursor Ion (Q1 Mass; Da)	Fragment Ion (Q3 Mass; Da)	Time (ms)	DP (V)	EP (V)	CE (V)	CXP (V)
FF1	356	336	100	−50	−5	−15	−8
FF2	356	185	100	−50	−5	−17	−12
FFA1	248	230	200	45	5	22	25
FFA2	248	130	200	45	2	30	10
CAF-d_5_ (IS)	326	157	100	25	10	−25	−20

FF1: florfenicol fragment ion 1, used as a quantifier ion; FF2: florfenicol fragment ion 2, used as a qualifier ion; FFA1: florfenicol amine fragment ion 1, used as a quantifier ion; FFA2: florfenicol amine fragment ion 2, used as a qualifier ion; CAF-d_5_: chloramphenicol-d_5_; IS: internal standard; Q1: quadrupole 1; Q3: quadrupole 3; DP: declustering potential; EP: entrance potential; CE: collision energy; CXP: collision cell exit potential.
